# Supplementary material for: Theobromine is associated with slower epigenetic ageing
Source: Aging (Albany NY). 2025 Dec 10;17(12):2902–15. doi: 10.18632/aging.206344 (PMC13147728; doi:10.18632/aging.206344)
Supplement: Supplementary Figure 1 [file aging-17-12-206344-s001.pdf]

## SUPPLEMENTARY FIGURE

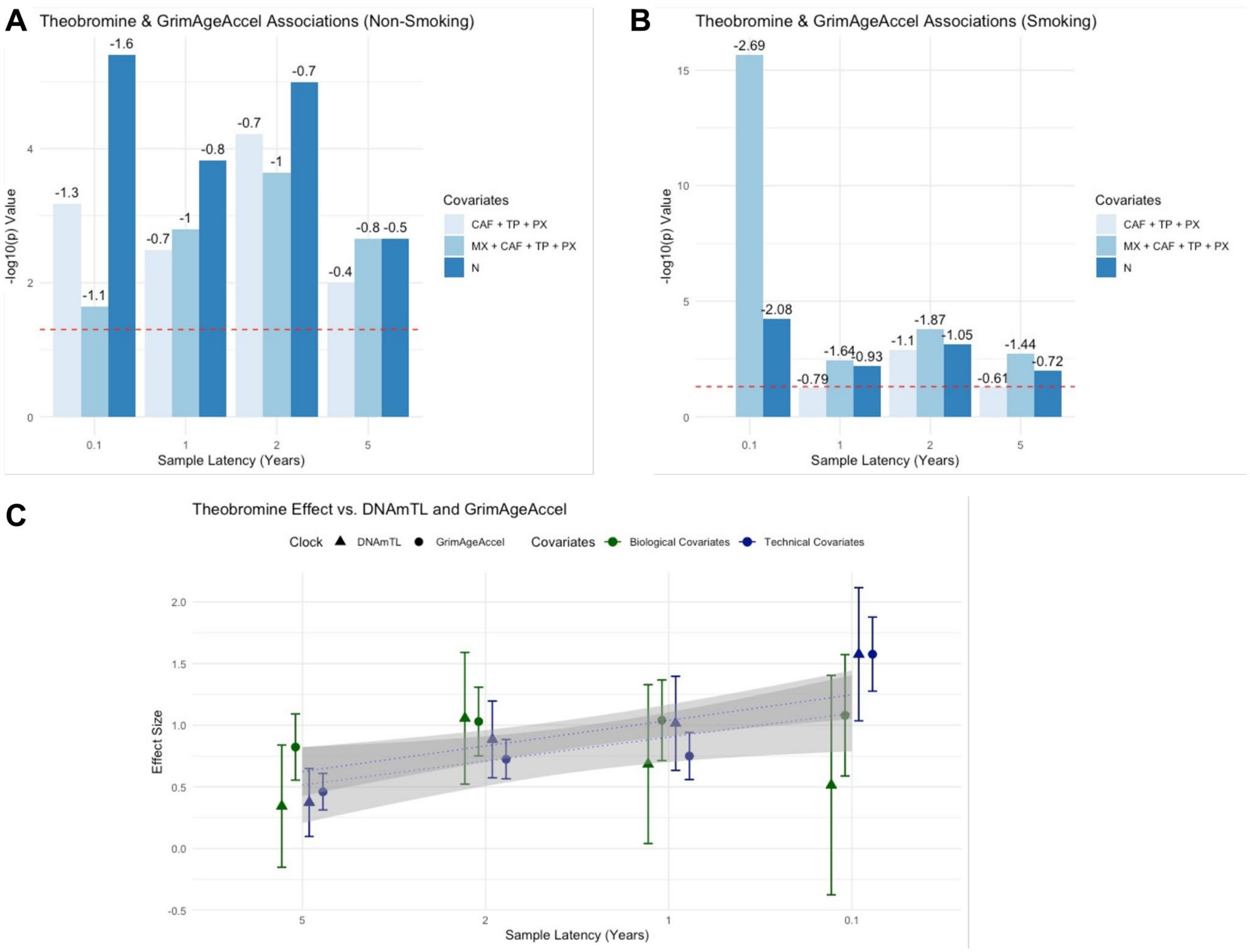


**Supplementary Figure 1. Sensitivity analyses exploring the specificity of the theobromine association with epigenetic ageing measures in the TwinsUK sample.** (**A**, **B**) Theobromine and GrimAge association significance in never smokers and current/ ex-smokers. Covariates in the models included 'N' (BMI + Age + Cell Proportions), as well as 'N' and methylxathines CAF, TP, PX and MX, as labelled on the Figure. TB coefficient effect sizes are shown. (**C**) Magnitude of effect sizes of DNAmTL association with GrimAgeAccel. As the scales and direction differ (with negative GrimAgeAcel and positive DNAmTL both indicating a slower age) the regression coefficient values of GrimAgeAccel were converted to absolute values and maximum values of DNAmTL were scaled to GrimAgeAccel (scale factor: 43.43). Correlation lines between latency values are applied (linear model) and standard errors are shown.
