# Supplementary material for: Theobromine is associated with slower epigenetic ageing
Source: Aging (Albany NY). 2025 Dec 10;17(12):2902–15. doi: 10.18632/aging.206344 (PMC13147728; doi:10.18632/aging.206344)
Supplement: Supplementary Table 1 [file aging-17-12-206344-s002.pdf]

Supplementary Table 1. Association results in TwinsUK cohort.

Sample Latency (yrs)	n	Covariates	GrimAge			DNAmTL			DunedinPACE			PhenoAge			AgeAccelHannum		
			β	*se*	*p*	*β*	*se*	*p*	*β*	*se*	*p*	*β*	*se*	*p*	*β*	*se*	*p*
5	509	N	-0.46	0.148352	0.002227	0.008597492	0.006367478	0.1784	0.002433033	0.003743138	0.5135	-0.1474319	0.2116257	0.4955	0.2210168	0.179496	0.2135
2	420	N	-0.724	0.1596351	1.03E-05	0.020360325	0.007167899	0.004462	-0.0068576	0.004158709	0.09612	-0.3780389	0.245318	0.128	0.03520191	0.20896333	0.8581
1	276	N	-0.75	0.1907382	0.00015	0.02338561	0.00878681	0.00798	-0.004733561	0.004859301	0.3249	-0.4952221	0.3012944	0.09743	-0.06744427	0.24238152	0.7961
0.1	121	N	-1.576	0.3005898	3.99E-06	0.03628296	0.01242632	0.002985	-0.007792432	0.006966381	0.3101	-0.3016093	0.4554014	0.5374	-0.04750247	0.33094191	0.918
5	509	CAF + TP + PX	-0.44	0.1698753	0.01004	0.010702108	0.007202701	0.1362	-0.001484194	0.004284681	0.7303	-0.1722435	0.2427985	0.4777	0.2069323	0.205579	0.3087
2	420	CAF + TP + PX	-0.734	0.1795713	6.07E-05	0.021952714	0.007920683	0.005354	-0.006831862	0.004684106	0.1399	-0.366313	0.2735349	0.1787	0.02361039	0.23384471	0.9211
1	276	CAF + TP + PX	-0.667	0.2215733	0.003227	0.02729603	0.01000793	0.006141	-0.001898739	0.005626773	0.7353	-0.4451804	0.3473743	0.1921	-0.1975919	0.2786004	0.4809
0.1	121	CAF + TP + PX	-1.274	0.3532336	0.0006571	0.0298907	0.01470864	0.03303	-0.001529166	0.007725097	0.86	-0.1889974	0.5272425	0.7243	-0.07701537	0.39117402	0.853
5	509	CAF + TP + PX + MX	-0.823	0.2680991	0.002185	0.007905393	0.011399421	0.4884	-0.001865078	0.006759327	0.7805	-0.08618605	0.37925441	0.8215	0.06192471	0.32195188	0.8439
2	420	CAF + TP + PX + MX	-1.03	0.2794238	0.0002277	0.02432609	0.01231021	0.04634	-0.008733971	0.007285852	0.2239	-0.1354184	0.4227936	0.7471	-0.2425322	0.3602748	0.4988
1	276	CAF + TP + PX + MX	-1.04	0.3276549	0.001572	0.01576093	0.01482524	0.2866	-0.003045039	0.008400915	0.7095	-0.01868127	0.51170186	0.9708	-0.1721211	0.4128901	0.6739
0.1	121	CAF + TP + PX + MX	-1.08	0.4919467	0.02253	0.01183208	0.02050152	0.5457	0.01806164	0.01014006	0.0739	0.1613058	0.7467483	0.802	-0.3171138	0.5498856	0.5286

Sample Latency is provided in years between methylation and metabolomic measurements. Covariates included in the linear model are ‘N’ (BMI + Age + Cell Proportions) and methylxathines CAF, TP, PX and MX as labelled.
